# Improvement in Asthma Symptoms and Pulmonary Function in Children After SARS-CoV-2 Outbreak

**DOI:** 10.3389/fped.2021.745611

**Published:** 2021-10-22

**Authors:** Jessica Taytard, Florence Coquelin, Nicole Beydon

**Affiliations:** ^1^AP–HP, Centre de Référence des Maladies Respiratoires Rares, Service de pneumologie pédiatrique, Hôpital Armand-Trousseau, Paris, France; ^2^Sorbonne Université, INSERM, UMRS1158, Paris, France; ^3^APHP, Unité Fonctionnelle de Physiologie-Explorations Fonctionnelles Respiratoires, hôpital Armand-Trousseau, Paris, France; ^4^INSERM U934, Centre de Recherche Saint Antoine, Paris, France

**Keywords:** asthma, lung function, lung function test, SARS-cov-2 outbreak, children

## Abstract

**Introduction:** Little is known on the effect of SARS-CoV-2 circulation on asthma daily symptoms in children. We compared asthma exacerbations, asthma symptom control and lung function before and after SARS-CoV-2 outbreak in children.

**Methods:** Retrospective study of children with persistent asthma referred for lung function testing. The second quarter of 2020 being a period with nearly no activity, we compared the activity between the first, third and fourth quarters of 2019 and 2020 (Q1-2019 vs. Q1-2020, Q3-2019 vs. Q3-2020 and Q4-2019 vs. Q4-2020).

**Results:** We retrieved 1,871 files in 2019 and 1,548 in 2020. The whole population [2,165 (63.3%) boys] had a median [IQR] age of 9.7 [6.8;13.1] years. There was no difference in age, sex, and ethnicity between 2019 and 2020 populations. Asthma was better controlled during Q4-2020 compared to Q4-2019 (*P* = 0.042), and there was a lower proportion of children with at least one exacerbation in the previous 3 months after the reopening, compared to the same period in 2019 (*P* < 0.0001). Baseline FEV_1_ (Z-score) recorded after the reopening was significantly higher (with less reversibility) compared to the same period before the epidemic (*P* < 0.0001). Baseline FEV_1_/FVC (Z-score) was significantly higher during Q3-2020 compared to Q3-2019 (*P* = 0.026), with fewer children having a significant reversibility (*P* = 0.035).

**Discussion:** We demonstrated a trend toward increased exacerbations just before the recognition of the epidemic, and fewer exacerbations, better asthma symptom control and improvement in the lung function of asthmatic children after the reopening.

## Introduction

Asthma symptoms and exacerbations are frequently secondary to viral respiratory infections but the capacity of SARS-CoV-2 to induce such symptoms is not fully known. The dramatic decrease in Emergency Room (ER) visits for asthma exacerbations in children following the SARS-CoV-2 outbreak may not entirely reflect a decrease in the occurrence of acute symptoms since families may have given up coming to the ER in order to minimize the risk of SARS-CoV-2 exposure (1–3). We wished to compare asthma exacerbations, asthma symptom control and lung function in children seen in our Lung Function Test (LFT) laboratory before and after the SARS-CoV-2 outbreak. Our hypothesis was that through social distancing, hygienic measures, and possible better adherence to asthma treatment, the SARS-CoV-2 pandemic would result in lower virus circulation, reduced asthma symptoms (including exacerbations) and improved lung function.

## Methods

We retrospectively retrieved from our database all files of children referred, from the asthma clinic of Trousseau University Hospital (Paris, France), for persistent asthma follow-up without comorbidities, except for cases of obesity. In order to take into account the variation of our activity during and after the first lockdown (no activity from mid-March to mid-May 2020, partial activity at the reopening from mid-May to end of June 2020, full activity thereafter including during a second, laxer lockdown from the end of October to mid-December 2020), we compared 2019 to 2020 activity levels excluding the second quarter of each year. The first quarter went from January to March, the second quarter from April to June, the third quarter from July to September and lastly the fourth quarter from October to December.

In our Standard Of Procedure (SOP), all asthmatic children and their families were seen by a physician before the LFT was performed. During this visit, a standardized questionnaire was filled out, including the gradation of asthma symptom control during the last month, and the occurrence of moderate or severe exacerbations in the previous 3 months (4, 5). As recommended, asthma symptom control, assessed using clinical items (night and daytime symptoms, reliever use, limitation of activities), was classified as well controlled, partly controlled or uncontrolled according to GINA recommendations (4). Moderate exacerbations were defined as acute asthma symptoms requiring at least 2 days in a row of reliever, and severe exacerbations as acute symptoms requiring at least 3 days of oral corticosteroids (5). We recorded reliable baseline and post-bronchodilator (Salbutamol 400 μg) spirometry tests according to international recommendations (6). Proportions of children and functional indexes (FEV_1_, FVC, and FEV_1_ reversibility) were compared using Chi-2 and unpaired t-Student tests or Mann-Whitney test, respectively, using GraphPad Prism v.6.07 software. *P* ≤ 0.05 was considered as significant. Parents gave oral consent for the possible retrospective use of their children's results and the institutional review board deemed the study exempted from further approval. The data base used in this study is declared to the French national data protection authorities (CNIL) which is aware of its possible retrospective use. Moreover, if parents refuse the use of their children's data, a box is checked in the data base which indicates this refusal. The children's files are then excluded from the data collection. In the present study no parents had refused the use of their children's data.

## Results

Asthma symptom control and exacerbations were reported in 3,166 (92.6%) and 3,062 (89.6%) out of 3,419 files retrieved (1,871 in 2019 and 1,548 in 2020), respectively. Nearly all patients where seen only once during the study period (3,394/3,419, 99.3%) The whole population comprised 1,254 (36.7%) girls and 2,165 boys (63.3%) with a median [IQR] age of 9.7 [6.8;13.1] years. There was no difference across years in age (median [IQR] age of 9.7 [6.9;13] and 9.9 [6.8;13.2] years in 2019 and 2020, respectively, *P* = 0.22), sex (36.8 vs. 36.6% girls, and 63.2 vs. 63.4% boys in 2019 and 2020, respectively, *P* = 0.89) and ethnicity (68.7 vs. 65% Caucasians, 18.8 vs. 21.4% Afro-Caribbean, 1.5 vs. 1.2% Asians in 2019 and 2020, respectively, *P* = 0.09). Obese and overweight children were 153 (4.5%) and 384 (11.2%), respectively, equally distributed across the six study quarters (*P* = 0.31).

**Figure 1:**
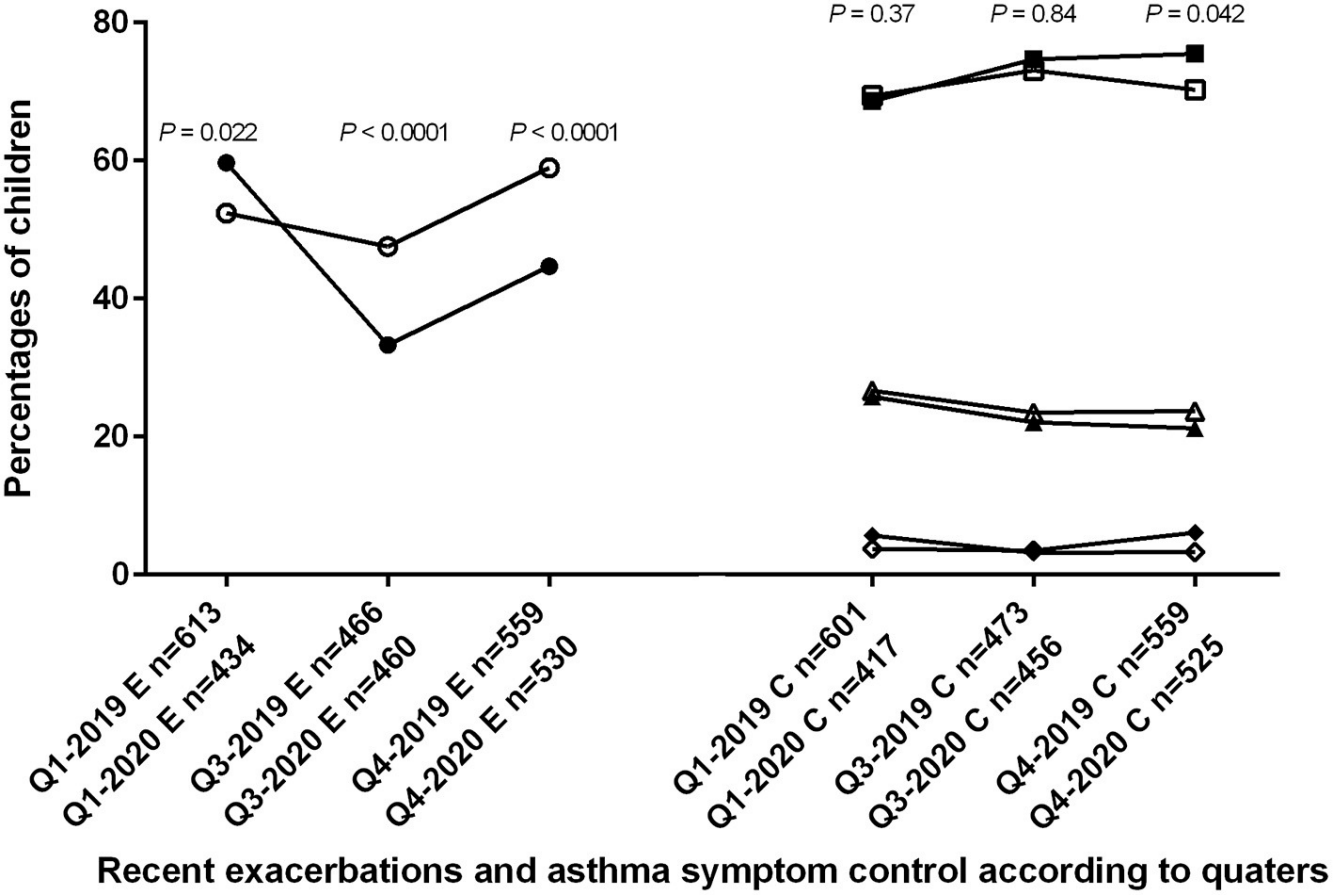
Trends in 2019 and 2020 for asthma exacerbations in 3,166 children, and for asthma symptom control in 3,062 children. Q1-2019, Q3-2019, Q4-2019, Q1-2020, Q3-2020, and Q4-2020: first, third and fourth quarters of 2019 and 2020, respectively; E: results concerning the proportion of children with exacerbations; C: results concerning the proportion of children with different level of asthma symptom control. Open symbols represent the year 2019 and closed symbols the year 2020. Circles are percentages of children with exacerbations. Percentage was significantly higher during Q1-2020 compared to Q1-2019, and significantly lower during Q3-2020 and Q4-2020 compared to Q3-2019 and Q4-2019, respectively. Squares are percentages of children with controlled asthma symptom; triangles are percentages of children with partially controlled asthma symptom; diamonds are percentages of children with uncontrolled asthma symptom. Control of asthma was significantly better only during Q4-2020 compared to Q4-2019.

Figure 1 shows the distribution of the 3,419 files across the first (Q1-2019 and Q1-2020), third (Q3-2019 and Q3-2020) and last (Q4-2019 and Q4-2020) quarters in 2019 and 2020, along with proportions of children with at least one recent exacerbation, and proportions of children with controlled, partially controlled or uncontrolled asthma symptoms.

The difference across the three classes of asthma symptom control was significant only between the last quarters of 2019 and 2020. Asthma was better controlled during Q4-2020 compared to Q4-2019 (for controlled, partially controlled and uncontrolled asthma 75.5, 21.2, and 3.3, vs. 70.3, 23.7, and 6.1%, respectively; *P* = 0.042) but similarly controlled during Q1-2019 and Q1-2020 (69.4, 26.7, and 3.8 vs. 68.6, 25.8, and 5.7%, respectively; *P* = 0.37), and during Q3-2019 and Q3-2020 (73.1, 23.5, and 3.5 vs. 74.7, 22.1, 3.2%, respectively; *P* = 0.84) (Figure 1).

Compared to 2019, the proportion of children with at least one exacerbation in the previous 3 months was higher in 2020 before the lockdown (Q1-2020 259/434 (59.7%) vs. Q1-2019 321/613 (52.4%); *P* = 0.022) but lower after the reopening (Q3-2020 153/460 (33.3%) and Q4-2020 237/530 (44.7%) vs. Q3-2019 222/466 (47.6%) and Q4-2019 330/559 (59%), respectively, both *P* < 0.0001) (Figure 1).

Table 1 shows the results of baseline and post-bronchodilator spirometry performed in 2,488/3,419 (72.8%) and 2,217/2,488 (89.1%) children, respectively. Baseline FEV_1_ (Z-score) recorded after the reopening was significantly higher compared to the same period before the epidemic (Q3-2020 FEV_1_ −0.03 vs. Q3-2019 −0.49, *P* < 0.0001, and Q4-2020 FEV_1_ −0.15 vs. Q4-2019 −0.29, *P* < 0.0001). Baseline Q3-2020 FEV_1_/FVC (Z-score) was also significantly higher compared to baseline Q3-2019 (−0.62 vs. −0.82, respectively; *P* = 0.026) with fewer children having a significant reversibility (i.e., FEV_1_ change ≥ 12% baseline) (12.9 vs. 18.8%, *P* = 0.035).

**Table 1:** Baseline (*n* = 2,488) and post-bronchodilator (*n* = 2,217) pulmonary function recorded in 2019 and in 2020, according to quarters.

**Results are median [IQR] or No. (%)**	**Q1-2019**	**Q1-2020**	**Q3-2019**	**Q3-2020^c^**	**Q4-2019**	**Q4-2020^d^**
**Baseline spirometry (*****n*** **=** **2,488)**						
FEV_1_ (Z-score)	−0.54 [−1.26;0.44]	−0.32 [−1.13;0.46]	−0.49 [−1.21;0.40]	−0.03 [−0.81;0.75]	−0.29 [−1.23;0.42]	−0.15 [−0.81;0.67]
FEV_1_/FVC (Z-score)	−0.81 [−1.63;0.02]	−0.88 [−1.60;0.09]	−0.82 [−1.55;0.11]	−0.62 [−1.36;0.01]	−0.72 [−1.53;0.04]	−0.53 [−1.50;0.18]
Bronchial obstruction^a^	120/485 (24.7)	79/339 (23.3)	88/391 (22.4)	71/368 (19.3)	112/482 (23.2)	85/422 (20.1)
**Post-bronchodilator spirometry (*****n*** **=** **2,217)**						
FEV_1_ reversibility (% baseline)	5.2 [1.2;10.1]	5.3 [1.8;9.7]	5.1 [1.7;10.1]	4.4 [1.8;8.3]	5 [2.0;9.0]	4.2 [0.9;8.9]
Significant reversibility^b^	82/442 (18.6)	50/289 (17.3)	68/362 (18.8)	42/326 (12.9)	68/433 (15.7)	50/365 (13.7)

*Q1, first quarter; Q3, third quarter; Q4,fourth quarter; FEV_1_, forced expiratory volume in 1 s; FVC, forced vital capacity*.

a *Bronchial obstruction defined as FEV_1_/FVC ≤ −1.645 Z-score*.

b *Significant reversibility defined as a FEV_1_ increase ≥ 12% baseline*.

c *Significant differences between Q3-2019 and Q3-2020 for FEV_1_ (P <0.0001), FEV_1_/FVC (P = 0.026), FEV_1_ reversibility (P = 0.039), and frequency of significant FEV_1_ reversibility (P = 0.035)*.

d *Significant differences between Q4-2019 and Q4-2020 for FEV_1_ (P <0.0001), and FEV_1_ reversibility (P = 0.031)*.

There was less FEV_1_ reversibility (% baseline) after the reopening during Q3 and Q4-2020 compared to the same time periods in 2019 (Q3-2020 4.4% vs. Q3-2019 5.1%, *P* = 0.039 and Q4-2020 4.2% vs. Q4-2019 5.0%, *P* = 0.031).

In 2019, we did not find that lung function (FEV_1_ Z-score, FEV_1_/FVC Z-score, and FEV_1_ reversibility % baseline) was significantly different across the three classes of asthma symptom control. In 2020, these results were similar for FEV_1_ Z-score and FEV_1_ reversibility % baseline, but FEV_1_/FVC Z-score significantly decreased with worse asthma symptom control (*P* = 0.02). Notably, the percentage of children with a significant FEV_1_ reversibility was significantly different between the three classes of asthma symptom control in 2019 and in 2020 (for controlled, partially controlled and uncontrolled asthma in 2019 14.7, 23.7, and 24.4%; *P* = 0.002, and in 2020 13.1, 18.7, and 35.3%; *P* = 0.0005, respectively).

## Discussion

This is the first study comparing, in a large population of asthmatic children of similar age, ethnicity and sex, the symptoms and the lung function before and after the SARS-CoV-2 outbreak. Previous studies have shown a dramatically decrease in ER visits for asthma exacerbations (1–3). However, data on asthma symptom control and on the frequency of home-based exacerbations during and immediately after the lockdown are scarce. Our study confirms results reported in smaller populations which showed a decrease in asthma exacerbations and an improvement in asthma symptom control during the SARS-CoV-2 outbreak (7, 8). We also present original concurrent results of improvement in lung function tests during the COVID-19 pandemic.

Asthma symptom control was more frequently achieved during the only studied quarter (Q4-2020) which included a period of lockdown for non-essential workers and for middle and high schools. This is concordant with a study in which asthma control scores from 85 children increased during the lockdown (9).

Also we found that, in 2019 and 2020, worse asthma control was statistically related to more frequent significant FEV_1_ reversibility. No relationship was found between asthma control status and other lung function indices during the study period. This result mirrors that of a recent study conducted in adult asthmatic patients in which significant bronchodilation was related to poor asthma control independently of the presence of baseline bronchial obstruction (10). Interestingly, the decrease in exacerbation rate after the first reopening corresponded to the period when children went back to school (September 2020). This significant reduction compared to the peak of asthma exacerbations usually seen during the fall could evoke lowered airway inflammation from reduced viral attacks and/or from better inhaled corticosteroids use. However, the exacerbation rate increased from Q3-2020 to Q4-2020 while asthma control improved. The difference between the periods of evaluation of asthma exacerbations (3 months before LFT) and asthma control (1 month before LFT) might explain this apparent discrepancy. Though this study focuses on LFT results, it would be interesting to compare the trends of exacerbations in asthmatic patients to the number of hospital admission during this period. A recent study showed that hospital admissions for asthma were significantly lower during lockdown, suggesting a reduction not only of mild, but also of severe exacerbations (9).

The low frequency of exacerbation concurred with an improvement in pulmonary function, in line with the known relationships between these two features of asthma (11, 12). While the degree of disease severity, as defined by the presence of bronchial obstruction, was similar in all quarters, continuous analysis of lung function indexes (baseline FEV_1_ and reversibility, FEV_1_/FVC) showed an improvement after the reopening. Various explanations could support this finding. First, the implementation of new hygienic measures and social distancing in all communities could have decreased the circulation of respiratory viruses. Second, the lockdown could have led patients to reduce their exposure to outdoors allergens and airborne pollution. Third, we cannot exclude a better adherence to asthma treatments following the recognition of the respiratory tropism of SARS-CoV-2. Lastly, despite a global decrease in exacerbations after the reopening, we still observed an increase in exacerbation rate during the last quarter compared to the third quarter in 2020 (Figure 1). As virus were probably less present in 2020, these exacerbations might have been triggered by indoors allergens or irritants (tobacco smoke exposure).

We acknowledge that this study has several limitations, the main ones being the declarative nature of data for asthma control and exacerbations collection, and the absence of precise information on patients' outdoors/indoors exposure during the lockdown. However, we used a standardized questionnaire to report acute symptoms of asthma and their treatments. Moreover, the improvement of asthma symptom control and lung function was recorded after the re-opening when exposure had returned to usual levels in our large city area. We also did not collect data on treatment adherence of which the evaluation by patients and families is questionable. Lastly, we were not able to show longitudinal LFT in the same patients, the frequency of LFT for each patient having decreased since the beginning of the pandemic. We compared the 2019 and 2020 populations in terms of age, sex and ethnicity and found no difference.

The follow-up of the relationships between asthma symptoms, pulmonary function and variable measures such as curfew or lockdown, could give further insights into the cause for good asthma outcomes.

## Data Availability Statement

The raw data supporting the conclusions of this article will be made available by the authors, without undue reservation.

## Author Contributions

JT, NB, and FC had full access to all of the data in the study and take responsibility for the integrity of the data and the accuracy of the data analysis. They are responsible for designing the study, data collection, analysis and interpretation, and drafting the manuscript. All authors contributed to the article and approved the submitted version.

## Conflict of Interest

The authors declare that the research was conducted in the absence of any commercial or financial relationships that could be construed as a potential conflict of interest.

## Publisher's Note

All claims expressed in this article are solely those of the authors and do not necessarily represent those of their affiliated organizations, or those of the publisher, the editors and the reviewers. Any product that may be evaluated in this article, or claim that may be made by its manufacturer, is not guaranteed or endorsed by the publisher.
